# Identifying Epidermolysis Bullosa Patient Needs and Perceived Treatment Benefits: An Explorative Study Using the Patient Benefit Index

**DOI:** 10.3390/jcm10245836

**Published:** 2021-12-13

**Authors:** Nicholas H. B. Schräder, Eva W. H. Korte, José C. Duipmans, Roy E. Stewart, Maria C. Bolling, André P. Wolff

**Affiliations:** 1 Department of Dermatology, University Medical Centre Groningen, University of Groningen, 9700 RB Groningen, The Netherlands; e.w.h.korte@umcg.nl (E.W.H.K.); j.c.duipmans@umcg.nl (J.C.D.); m.c.bolling@umcg.nl (M.C.B.); 2 Department of Public Health, University Medical Centre Groningen, University of Groningen, 9700 RB Groningen, The Netherlands; r.e.stewart@umcg.nl; 3 Anaesthesiology Pain Centre, University Medical Centre Groningen, University of Groningen, 9700 RB Groningen, The Netherlands; a.p.wolff@umcg.nl

**Keywords:** epidermolysis bullosa, patient perspectives, patient benefit index, clinical outcomes

## Abstract

Epidermolysis bullosa (EB) is a genetic blistering skin condition for which no cure exists. Symptom alleviation and quality of life are therefore central to EB care. This study aimed to gain insight into EB patient needs and benefits from current clinical care. Two questionnaires were administered cross-sectionally to adult EB patients at the Dutch expertise centre for blistering diseases. Patient needs and benefits were analyzed using the patient benefit index survey (PBI-S). Ancillary data were compiled pertaining to self-reported EB severity, pain and pruritus, as well as current and previous treatments. In total, 104 participants were included (response rate 69.8%). Sixty-eight participants comprised the analyzed cohort (*n* = 36 omitted from analysis). The needs given the highest importance were *to get better skin quickly* (64.7%) and *to be healed of all skin alterations* (61.8%). A positive correlation between pain and EB severity and the importance of most needs was observed. Minimal clinically important differences within the PBI-S, relating to reported benefits from clinical care, were reported by 60.3% of the cohort. This study highlights a discrepancy between patient needs and feasible treatment outcomes. Utilizing the PBI-S in conjunction with well-established multidisciplinary care may catalyze the process of tailoring treatments to the needs of individual patients.

## 1. Introduction

Epidermolysis bullosa (EB) is an inherited skin condition with known variants in 16 genes and comprises a heterogenous group of clinical phenotypes. The main types are based on the level of blister formation and include EB simplex (EBS), junctional EB (JEB), dominant dystrophic EB (DDEB), recessive dystrophic EB (RDEB) and Kindler EB [1].

EB is characterised by blistering of the skin and mucous membranes, with some forms showing extracutaneous involvement, either secondary to chronic extensive wounds and scarring, or as a direct consequence of molecular defects [2,3]. 

Patients report significant effects on their quality of life (QoL) due to chronic symptoms, incurred treatment costs, reduced work/school productivity and low mood-states, amongst an array of factors [4,5,6,7,8,9]. Complications, such as aberrant wound healing, scarring and infection, as well as the psychosocial impact, mean that this disease is clinically challenging and requires expert multidisciplinary management.

As EB cannot be cured, numerous clinical practice guidelines are aimed at the prevention of secondary pathologies, symptom alleviation and psychosocial improvements. These are presently the most tangible elements for improving day-to-day QoL of EB patients [10,11,12,13]. In order to effectively improve outcomes and the QoL of EB patients, it is imperative that clinicians pay adequate attention to the needs in both individual patients and distinct EB types [14]. This study aimed to gain insight into EB patient needs and their reported benefits from their current EB care. Additional demographics, patient-reported characteristics, pain and pruritus treatments and recreational drug-use were taken into account.

## 2. Materials and Methods

This was a cross-sectional study at the European Reference Network (ERN) expertise centre for blistering diseases in the University Medical Centre Groningen (UMCG) [15]. Participants had a genetic diagnosis of inherited EB, were at least 16 years old, were living in the Netherlands, could read and write in Dutch and could provide informed consent. Eligible participants were identified in the Dutch EB registry, contacted by telephone, informed about the study procedures and invited to participate through the dispensation of a brochure and consent form. The Dutch EB registry does not discriminate between patients receiving treatment at the UMCG or other healthcare settings; therefore, the latter were excluded from analysis after data collection. The study was approved by the UMCG institutional ethics review board on 8 January 2019 (METc #201800968).

Participants were administered two surveys. Part A was a self-assembled survey on patient burden of illness and treatments, completed through a telephone interview. Burden of illness comprised self-reported EB severity (mild, moderate and severe), as well as pain and pruritus intensity throughout the previous week (numeric rating scale (NRS): 0–10 for morning, afternoon and evening). Participants reported prescribed pharmacologic pain and pruritus treatments, used at the time of survey completion. Systemic pharmacologic treatments that were previously prescribed were reported using a multiple-choice list based on systemic therapies from best practice EB guidelines [10]. Lastly, recreational drug-use (tobacco, alcohol, cannabis and other drugs) and its effects on EB were reported. Responses were categorized during post-hoc analysis.

Part B consisted of the validated patient benefit index survey (PBI-S), standard version, completed digitally by participants. The PBI-S permitted the evaluation of individual needs and benefits in the treatment of dermatologic conditions [16]. The first section was comprised of the patient needs questionnaire (PNQ). Participants rated the importance of each of the 25 statements (not at all, somewhat, moderately, quite, very and does not apply to me). The second section, the patient benefit questionnaire (PBQ), consisted of the same 25 statements. However, participants reported the extent to which the PNQ statements were achieved. In this study, participants were asked to refer to their “general EB clinical care at the UMCG”. The combined PNQ and PBQ data enabled the calculation of the patient benefit index (PBI). The PBI could range from no (0) to maximum benefit (4). A PBI score ≥ 1 portrayed a minimal clinically important difference (MCID) after intervention, taken from PBI validation studies in other dermatologic conditions [16]. The PBI-S distinguished between 5 subscales: physical impairments, psychological impairments, social impairments, impairments due to therapy and having confidence in healing [17].

Both surveys were built into a digital outcome monitoring program (RoQua). Sociodemographic and medical data (EB type, age, sex and time since diagnosis) were obtained from the Dutch EB registry [15]. Data were represented using descriptive statistics (frequencies and proportions for categorical data—means and standard deviations (SD), median values and interquartile ranges (IQR) for continuous data). Means comparisons were performed for normally distributed variables; otherwise, non-parametric testing was applied. Participants’ needs (PNQ) were correlated using the Spearman rho correlation coefficient, with self-reported outcomes (EB severity, pain and pruritus scores) and sociodemographic data (age and time since diagnosis). Bootstrapping was performed for confidence intervals. The correlation coefficients (rs) were defined as: negligible (0–0.29), low (0.3–0.49), moderate (0.5–0.69) and high (0.7–1.0) [18]. A two-sided *p*  =  0.05 was set a priori for statistically significant differences. Statistical analyses were performed using IBM SPSS Statistics (Version 23.0. Armonk, NY: IBM Corp.) and Stata Statistical Software (Release 16. College Station, TX, USA: StataCorp LLC.).

## 3. Results

### 3.1. Study Population

In total, 149 patients were contacted, of which 10 declined participation. A total of 104 participants returned either paper or electronically signed informed consent forms and completed both the PBI-S and supplementary survey (response rate: 69.8%). Subsequently, 36 participants were omitted from analysis due to scores of 0 on all PBQ items—these participants were not receiving treatment from the Dutch expertise centre for blistering diseases. Sixty-eight participants made up the study population (Table 1). The mean age was 41.1 years (SD ± 16.4), and mean time since diagnosis was 15.3 years (SD ± 8.0). There were 38 (55.9%) males and 30 (44.1%) females. Twenty-nine (42.6%) participants had EBS, ten (14.7%) had JEB, twenty-three (33.8%) had DDEB and six (8.8%) had RDEB [1].

### 3.2. Disease Burden

Self-reported EB-severities were mild (54.4%), moderate (29.4%) and severe (16.2%) (Table 1). Most RDEB participants (66.7%) reported having *severe* EB, followed by JEB (50.0%), DDEB (4.3%) and EBS (3.5%). The majority of participants with DDEB (65.2%) and EBS (51.7%) and all participants with JEB and RDEB had pain. The prevalence of pruritus was high in all EB types (EBS: 58.6%, JEB: 80.0%, DDEB: 82.6% and RDEB: 100%). In the total cohort, a minority of participants were entirely pain-free (*n* = 22, 32.4%) and pruritus-free (*n* = 18, 26.5%).

### 3.3. Treatments

Fewer than half of the participants (47.1%) were using prescribed pharmacologic treatments for pain (Table 2). The most prevalent drug treatment classes for pain were first and second-line analgesics—paracetamol (35.1%) and non-steroidal anti-inflammatory drugs (NSAID) (11.8%). Most DDEB (65.2%) and RDEB (66.7%) patients and half of JEB participants (50.0%) used at least one analgesic drug, whereas not many EBS participants did (27.6%). Only one quarter of all participants reported using pharmacologic pruritus treatments, of which antihistamines (66.7%) were the most prevalent. Of the previously used drug classes for pain and/or pruritus, paracetamol (80.9%), NSAIDs (67.6%), opioids (41.2%) and antihistamines (36.8%) were the most prevalent (Table 2). Twenty-five participants (36.8%) reported having previously not used any of the drug classes listed.

### 3.4. Recreational Drug-Use

Alcohol was the most prevalent recreational drug used (72.1%) and improved symptoms (mostly pain) in seven (14.3%) participants; however, alcohol worsened symptoms (mostly pruritus) in four (8.2%) participants (Table 2). Tobacco-use was less prevalent (17.6%), and no effects on EB-symptoms were reported. Cannabis was used by eight (11.8%) participants, of which five reported symptom reduction. In the total cohort, a minority reported not using any of the listed recreational drugs (20.6%).

### 3.5. Patient Benefit Index

The global patient benefit index (PBI) median was 1.34 (IQR: 0.68–2.58, range: 0–4) (Table 3). In 41 participants (60.3%), the global PBI was ≥1, indicating that at least a minimal clinically important difference (MCID) from treatment was achieved. The highest proportion of participants achieving an MCID was observed in JEB (80%), followed by RDEB (66.7%) and DDEB (60.9%). Less than half of EBS participants (48.3%) surpassed this threshold. MCID proportions in male (60%) and female (60.5%) participants were comparable. Significant differences in PBI scores between EB types and sex were not found, nor were any differences observed between the five PBI subscales (Table 3 and Table S1).

### 3.6. Patient Needs

**Figure 1:**
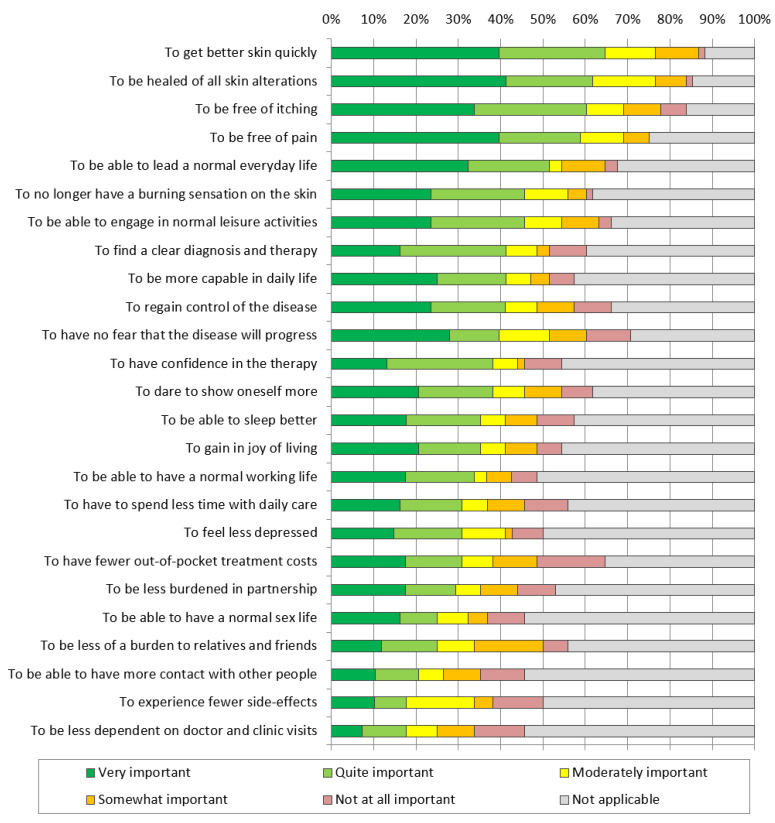
Patient needs questionnaire (PNQ) item importance in all participants in the total cohort (*n* = 68). Items are ordered by proportions of high importance (very important and quite important). Additional data can be found in the supporting information available online (Table S1).

The needs given the highest importance rating (combined percentage of *quite* and *very important*) were *to get better skin quickly* (64.7%) followed by *to be healed of all skin alterations* (61.8%) (Figure 1, Table S2). The highest importance assigned to needs related to a reduction in symptoms were *to be free of itching* (60.3%), *to be free of pain* (58.8%) and *to no longer have a burning sensation on the skin* (45.6%). Of lowest importance were the needs *to be less dependent on doctor and clinic visits* (17.6%) and *to have fewer side effects* (17.6%). 

PNQ-items with high importance were stratified by EB type (Table S2). All RDEB participants gave a high importance rating to the PNQ-items *to be free of itching* (100%) and *to be healed of all skin alterations* (100%). These items were also given the highest importance rating by DDEB participants (*to be healed of all skin alterations* (73.9%), *to be free of itching* (69.6%)). EBS and JEB participants reported the same two needs with highest importance; *to be free of pain* (EBS: 69.0%, JEB: 70.0%) and *to get better skin quickly* (EBS: 58.6%, JEB: 80.0%).

**Figure 2:**
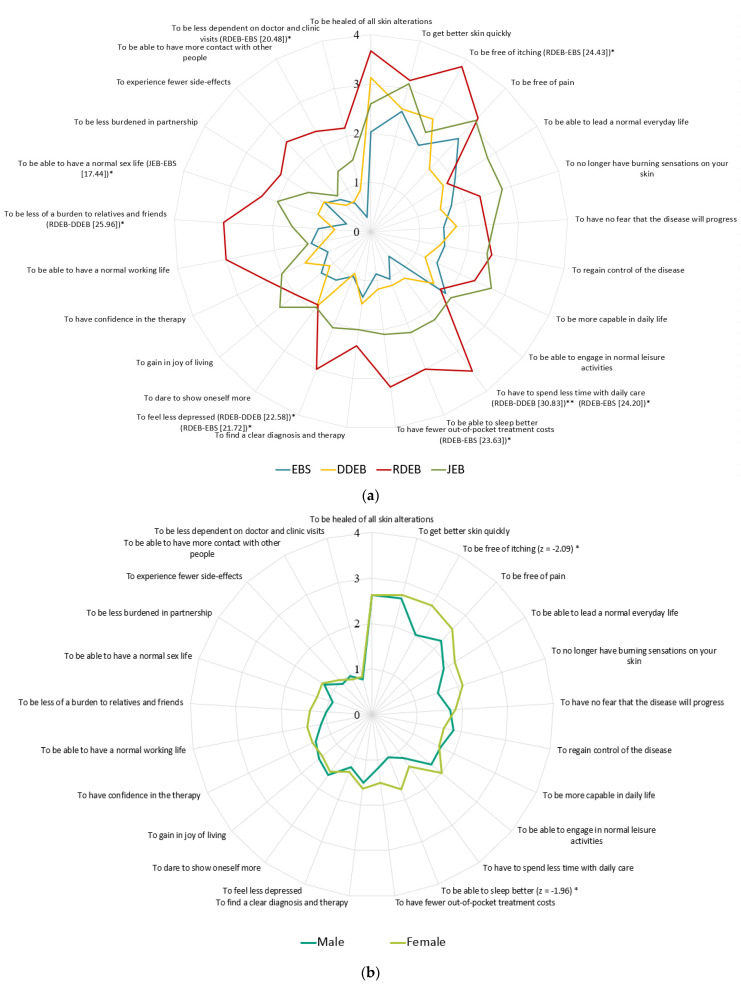
(**a**) Patient needs questionnaire (PNQ) items representing mean scores in each EB-subtype, in *n* = 68 participants. Chi-square values are shown for items with significant differences between types (EBS, RDEB, DDEB and JEB). (**b**) PNQ-items representing mean scores in each sex. Z-scores are shown for items with significant differences between sex (male and female). Items from A and B are listed in order of high importance in the total cohort. * (*p* < 0.05), ** (*p* < 0.01).

Pairwise comparisons across EB types revealed differences in seven PNQ-items (Figure 2a, Table S2). RDEB participants assigned a higher importance than EBS to the following items: *to be free of itching* (*Z* = −2.80, 95% Confidence Interval [CI], (0.004–0.006)), *to feel less depressed* (*Z* = −2.71, (0.004–0.007)), *to be less dependent on doctor and clinic visits* (*Z* = −2.85, [0.002–0.004]), *to have to spend less time with daily care* (*Z* = −3.92, (0.000–0.000)) and *to have fewer out of pocket treatment costs* (*Z* = −2.91, (0.001–0.003)). The largest differences between participants with RDEB and DDEB were for the following items: *to have to spend less time with daily care* (*Z* = −3.02, (0.000–0.002)), *to be less of a burden to relatives and friends* (*Z*= −2.87, (0.002–0.005]) and *to feel less depressed* (*Z* = −2.87, (0.003–0.006)). JEB participants reported a higher importance for the item *to have a normal sex life,* than EBS participants (*Z* = −2.98, (0.002–0.004)).

An exploration of pairwise differences in PNQ-item importance in all participants revealed significant differences between sexes (Figure 2b). Females reported higher importance ratings for *to be free of itching* (*Z* = −2.09, (0.031–0.038)) and *to be able to sleep better* (*Z* = −1.96, (0.040–0.048)). 

**Figure 3:**
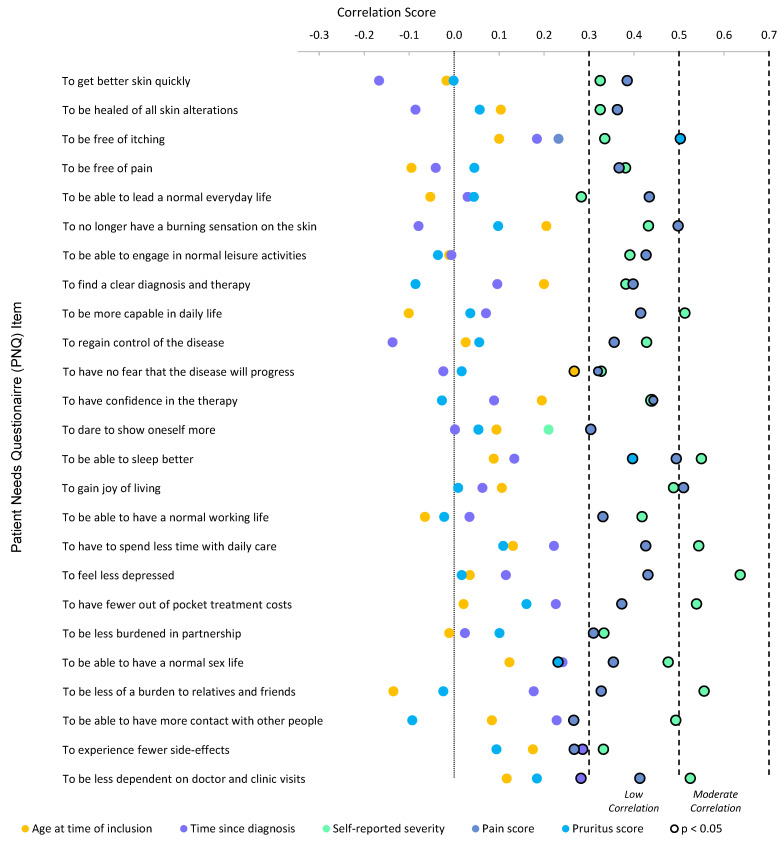
Scatter diagram in total cohort (*n* = 68) showing correlation scores of patient-reported outcomes (pain score; pruritus score; self-reported severity) and demographic data (age at time of inclusion; time since diagnosis) with 25 patient needs questionnaire (PNQ) items. The higher the correlation score, the more positive the correlation. Statistical significance is set to *p* < 0.05 (see figure legend). Additional data can be found in the supporting information available online (Table S3).

Participant-reported disease burden and demographic correlation analysis revealed several positive correlations with PNQ-item importance (Figure 3). Self-reported EB severity moderately correlated with seven PNQ-items, meaning that the more severe participants perceived their EB to be, the more important these needs became. The strongest correlation of self-reported severity was with the item *to feel less depressed* (r_s_ = 0.636, 95% Confidence Interval [CI] (0.443–0.801)). A moderate correlation was observed between pain scores and the PNQ-item *to gain joy of living* (r_s_ = 0.510 [0.317–0.660]). For pruritus scores, one moderate correlation was observed (*to be free of itching:* r_s_ = 0.503 (0.289–0.671)). All correlation scores for age and time since diagnosis were either insignificant or negligible.

## 4. Discussion

### 4.1. Burden of Disease

EB comprises a spectrum of disease-severities, of which RDEB and JEB generally make up the most severe EB types [9,19]. In this study, participants with RDEB and JEB reported the highest disease severity, as well as the highest prevalence of pain. By contrast, most EBS and DDEB participants reported a mild severity, and fewer EBS and DDEB participants experienced pain. Pruritus was reported by the majority of participants for each EB type. The highest and lowest prevalence and intensity of pruritus was reported by RDEB and EBS, respectively. Pain and pruritus are by nature complex symptoms of EB, with overlapping aetiologies. Both symptoms can stem from skin or mucosal wounds, inflammation and disrupted epidermal nerve fibres (neuropathies) [10,20,21,22]. Due to ongoing nociceptive and neuropathic pain states, as well as prolonged inflammation in EB, it is likely that nociplasticity occurs [23]. Recalcitrant pain and pruritus are the combined results of pathophysiological processes, the environment and psychosocial factors, which impact the quality of life of EB patients. These symptoms are therefore key determinants of the burden of having EB, because of which the further elucidation of their respective pathoaetiologies deserves the best research-oriented and clinical attention [10,19,21,24,25,26,27,28].

### 4.2. Therapies

Despite ongoing research, definitive cures have not yet been found for EB [29,30,31]. Enhancing quality of life is therefore important and can be further improved by understanding the needs of patients living with this disease. It is well known that the most beneficial approach to the symptomatic treatment of EB includes pharmacologic treatment as *one* of the treatment modalities. However, potential benefits are too often overshadowed by limited effectiveness and burdensome side-effects. Concomitant wound/protective dressings, indifferent topical therapy and psychosocial interventions must therefore be used synergistically as the cornerstone of EB treatment [10,32]. Although we observed a high prevalence of pain and pruritus, only three quarters of our participants were currently receiving analgesic or antipruritic pharmacologic therapies. In this cohort, many patients discontinued these treatments. This is likely due to inadequate effects, intolerance to side-effects or the fact that participants only used these treatments for short-term periods. However, based on the collected data, it was not possible to further examine the reasons for discontinuation.

Interestingly, a large discrepancy was seen in the prevalence of previous (*n* = 28) versus current (*n* = 3) opioid-use. Whilst opioids are not first-line analgesics in EB, they are modalities for severe acute and chronic pain in EB. Their limited effectiveness and side-effect profiles, including constipation, cognitive impairment, hyperalgesia and pruritus, as well as the risk for tolerance and dependence, may explain the observed discontinuation [33,34].

### 4.3. Recreational Drug-Use

The use of recreational drugs that entail significant health-risks has not yet been assessed in relation to EB. The collection of this data was exploratory and aimed to identify behaviours that may hinder symptom resolution in EB. The minority of participants using tobacco reported no effects on EB symptoms; however, the deleterious effects of tobacco on skin integrity, wound healing and neoplasms are well known [35,36]. Alcohol was the most prevalently consumed drug, because of which a small but noteworthy number of participants reported pain-reduction and pruritus-worsening. This is an important finding as evidence shows that alcohol consumption can induce clinically relevant pain reduction [37]. Therefore its consumption can be motivated by chronic pain and can perhaps demonstrate an inability to find effective analgesia in conventional treatment settings, despite the potential health consequences [38]. For that reason, the persistence of ineffective treatments in EB underpins the need to gain a better understanding of the risks of these behaviours and emphasizes the importance of incorporating appropriate counselling into regular clinical follow-up.

Interestingly, participants in this study reported symptomatic alleviation from cannabis-use. Given the reported benefits of cannabinoid-based medicines (CBMs) in recent studies, it is likely that these participants were self-medicating. The use of cannabinoid-based substances outside the controlled clinical setting outweighed prescribed CBMs in this cohort, which is likely due to the lack of cost reimbursement in the Netherlands. CBMs are gaining traction as potential therapies in EB; two case-series, as well as a recent international survey, highlight a plethora of patient-reported benefits from CBMs [39,40,41,42]. It is therefore imperative that new, high-quality research should ascertain the potential risks and benefits of CBMs in EB care.

### 4.4. Patient Benefit Index (PBI)

The majority of participants achieved the PBI threshold ≥ 1 for an MCID. Surprisingly, EBS was the only EB type whose MCID proportion was less than half. EBS participants reported the lowest burden of pain and pruritus, highest proportion of mild EB and lowest importance ratings for the PNQ-items. Although EBS is often considered mild, this may not mean that their needs are more readily met. In fact, milder clinical presentations and subjective disease experiences imply that the window of opportunity for meaningful outcome improvements in EBS is smaller. By contrast, a recent study highlighted unmet pain treatment needs in EBS that significantly impacted overall QoL [22]. These unmet treatment needs and benefits in EBS patients warrant further investigation to ascertain the observed pitfalls in recognizing and fulfilling EBS care needs. Utilizing the PBI-S may add value to clinical decisions based on patient-relevant needs.

### 4.5. Patient Needs: Items

The most important needs identified in this study were within the physical manifestations of EB (*to get better skin quickly* and *to be healed of all skin alterations*). Promising therapeutic research continually addresses underlying genetic defects, skin integrity, fibrosis and neoplasms; however, the findings in this study may reflect high patient expectations that, to date, are still unattainable in EB patient care [30,43,44,45]. It is critical that clinicians address the discrepancy between feasible treatment goals and patient expectations in EB, through adequate, repeated patient counselling, as existing evidence for other conditions show that expectation management improves perceived outcomes [46,47,48].

The significant burden of symptoms in EB is well known, and this was reflected in our study as symptomatic needs in all EB types were ranked highly (*to be free of pain, to be free of itching* and *to no longer have a burning sensation on the skin*). Unlike the physical manifestations of EB, symptoms such as pain and pruritus tend to be more tangible therapeutic targets. However, even with numerous treatment modalities, adequate symptomatic alleviation is a challenging feat. As a result, patients often develop coping strategies in a desperate attempt to manage these symptoms [14]. 

RDEB participants reported the highest frequency and importance for all needs. RDEB is characterized as a severe EB-type, with a high symptomatic burden and excessive home-based and clinical care needs [6,7,49]. In this study, this was reflected in the higher frequency and importance assigned to the following needs by RDEB participants: *to have to spend less time with daily care, to be less of a burden to relatives/friends, to have fewer out of pocket treatment costs* and *to be less dependent on clinical visits*. These same PNQ-items were less important for all other EB-types, thus distinguishing RDEB-needs; minimizing the care-related burden in RDEB remains a priority.

Previous studies have shown differences in depressive symptoms between DEB (RDEB and DDEB together) and control groups [25,50]. However, in this study the item *to feel less depressed* was significantly more important for RDEB than for DDEB participants. Depressive symptoms in RDEB should be given adequate clinical attention, as these may worsen the perceived pain, and vice versa [51]. Furthermore, subdividing DEB into DDEB and RDEB when assessing depressive symptoms in research settings is imperative. 

The PNQ analysis also highlighted more obscure patient needs, such as *to be able to have a normal sex-life*, which was significantly more important in JEB compared to EBS. Sexuality in relation to EB is underrepresented in the scientific literature; however, new international EB consensus guidelines on sexuality have been published [13,52].

Two PNQ-items, *to be free of itching* and *to be able to sleep better,* were significantly more important for females than males. Similar differences in the burden of pruritus between sexes in EB has been observed [4,53]. In other pruritic diseases, females report higher intensity scores and impact on QoL than men, which is likely exacerbated by emotional and psychosomatic factors [54]. Therefore, sex differences and emotional burdens in EB, especially in females, should be considered during pruritus oriented EB consultations. The negative effects of pruritus on quality of sleep are well described in other conditions, which may explain why females, with a higher need to reduce itching, also assigned a higher importance to the need related to sleep [55]. 

### 4.6. Patient Needs: Correlations

The correlation analysis revealed that a higher self-reported EB severity, irrespective of EB type, correlated with higher importance of needs in all but two PNQ-items. Patients’ perception of the severity of their EB is shaped by physical, sociodemographic, psychological factors, coping mechanisms and expectations [56]. Utilizing self-reported severity measurements awards clinicians with additional insight into the perceived disease burden, and subsequent individualised needs assessments further provide a backbone to formulating treatment goals. The needs *to dare to show oneself more* and *to be able to lead a normal everyday life* did not correlate with self-reported severity. This indicates that psychosocial support related to self-image and well-being is equally necessary for any EB severity, and is in line with recent EB guidelines on psychosocial support [13]. Using a structured survey such as the PBI can enhance clinical care attuned to psychosocial needs.

Pain scores correlated well with the majority of PNQ-items, which suggests the far reaching consequences of pain on daily life in EB [4,10]. By contrast, pruritus scores only correlated with two needs: *to be free of itching* and *to be able to sleep better.* Sparse research has assessed sleep quality in EB; however, one study observed disturbed sleeping patterns in DEB patients [57]. Other studies report that any EB type is at risk of negatively affected sleep quality [19,26,58]. These findings indicate a bidirectional interplay of sleep quality and symptoms, whereby improving either factor will positively affect the other. Even though the prevalence of pruritus in this cohort was high, the use of sedative antihistamine and anxiolytic medications, conventionally used to improve pruritus and aid sleep, was low, which may be due to their minimal effectiveness for pruritus in EB.

The absence of correlations between age and PNQ-items is an important finding, indicating that age does not influence adult EB-patient needs. As patients get older, it is unclear if their ability to deal with the physical and psychosocial consequences of EB improves. One study, however, observed correlations between stress and age, suggest that, as time goes by, patients adapt to the complications of their EB [59]. Additionally, in non-EB chronic pain, the relationship between pain and disability is more pronounced in younger people, and meta-analyses demonstrate dynamic age-related pain tolerance [60,61,62]. Although an age-to-needs relationship was not observed in this study, future research assessing the natural history of symptoms in EB could add important value to current paradigms in EB care.

### 4.7. Strenghts and Limitations

To the best of our knowledge, this is the first study of EB to implement a survey that assesses patient benefit outcomes weighted by individual needs. The results in this study complement existing qualitative and cross-sectional research on patient needs and consequences of living with EB [6,9,26,63]. The response rate in this study was high compared to other cross-sectional EB studies in our centre and demonstrates participants’ willingness to be involved in research that addresses their individual needs [4,24,26]. 

The PBI-S is designed to be implemented before and after an intervention. In our explorative design we omitted the assessment of a specific treatment in favour of “general EB clinical care at the UMCG”. This resulted in PBI scores not representing benefits from any single intervention. Additionally, participants were not pre-conditioned to score their needs based on “ideal” versus “realistic” expectations from EB care interventions, which means that some may have accounted for this when others did not. This may partially explain the low proportions of MCID achieved by participants. However, the limited effectiveness of conventional treatments still appears to be an important factor leading to inadequate treatment effectiveness and persistence of unmet needs in EB.

Importantly, we were unable to mitigate certain biases due to the retrospective nature of benefit reporting in the PBQ and pain/pruritus NRS. A recall bias may have been present when patients reported benefits, previous treatments and symptom scores. As recreational drug-use is a sensitive topic, patients were encouraged to be transparent when reporting; however, underreporting should be considered. Additionally the present state effect bias (how a participant feels at the moment of survey completion) is likely to have influenced the survey results; therefore, participants may have over- or under-reported their treatment benefits [64]. 

Although the PBI-S has been validated for general dermatologic conditions, we expect that it overlooks treatment goals unique to EB. Additionally, the PBI is limited in its assessment of benefits, insofar that it cannot assess worsening of symptoms or distancing from treatment goals due to therapy. Future studies should validate EB-specific PBI items through a combined patient and clinician consensus [65,66,67,68]. 

## 5. Conclusions

Effective treatments for EB remain elusive, and patient needs are not readily met. 

This study highlights an important discrepancy between patient needs and the feasible capabilities of the available treatments. The role of patient expectation management through transparent, informed and compassionate communication should be emphasized by healthcare-providers in the clinical setting in order to improve perceived treatment outcomes in EB. Utilizing outcome tools such as the PBI-S in conjunction with well-established multidisciplinary care may catalyze the process of tailoring treatments to the needs of individuals with EB. Furthermore, these results emphasize the continued need for developing effective treatments for EB and its accompanying symptoms in order to improve patients’ quality of life.

## Figures and Tables

**Table 1:** Demographic and patient-reported characteristics.

	All	EBS	JEB	DDEB	RDEB
Participants, n (%)	68 (100.0)	29 (42.6)	10 (14.7)	23 (33.8)	6 (8.8)
Age, mean ± SD	41.1 ± 16.4	39.7 ± 15.4	49.3 ± 19.4	41.5 ± 16.8	33.2 ± 12.5
Time since diagnosis (years), mean ± SD	15.3 ± 8.0	12.8 ± 7.4	19.6 ± 5.9	16.1 ± 7.9	16.7 ± 11.3
Sex, n (%)					
Female	38 (55.9)	17 (58.6)	4 (40.0)	13 (56.5)	4 (66.7)
Male	30 (44.1)	12 (41.4)	6 (60.0)	10 (43.5)	2 (33.3)
Presence of pain, n (%)	46 (67.6)	15 (51.7)	10 (100)	15 (65.2)	6 (100)
Presence of pruritus, n (%)	50 (73.5)	17 (58.6)	8 (80)	19 (82.6)	6 (100)
Pain NRS, median (IQR)					
Morning	2.0 (0.0–5.0)	0.0 (0.0–2.5) *	6.0 (5.0–7.0) *^,^**	2.0 (0.0–3.0) **	2.5 (1.0–7.0)
Afternoon	2.0 (0.0–5.0)	0.0 (0.0–2.0) *	5.0 (3.0–7.3) *^,^**	2.0 (0.0–4.0) **	3.0 (1.0–6.5)
Evening	2.0 (0.0–6.0)	0.0 (0.0–2.5) *	6.0 (5.0–7.3) *^,^**	2.0 (0.0–6.0) **	4.0 (1.0–7.0)
Mean all-day	2.0 (0.0–5.0)	0.7 (0.0–2.0) *	5.8 (4.8–6.6) *^,^**	1.3 (0.0–4.3) **	3.2 (1.0–7.1)
Pruritus NRS, median (IQR)					
Morning	1.0 (0.0–3.5)	0.0 (0.0–2.0) *	1.5 (0.0–4.3)	1.0 (0.0–4.0)	4.5 (2.5–7.0) *
Afternoon	1.0 (0.0–3.5)	0.0 (0.0–2.0) *	1.0 (0.0–3.3)	1.0 (1.0–4.0)	3.5 (3.0–5.8) *
Evening	2.0 (0.0–6.0)	1.0 (0.0–4.0)	5.5 (0.8–7.0)	3.0 (1.0–6.0)	5.0 (2.8–7.5)
Mean all-day	1.7 (0.0–4.0)	0.7 (0.0–2.3) *	2.8 (0.5–4.1)	2.0 (1.0–4.6)	4.7 (2.6–6.3) *
Self-reported Severity, n (%)					
Mild	37 (54.4)	19 (65.5)	2 (20.0)	16 (69.6)	0 (0.0)
Moderate	20 (29.4)	9 (31.0)	3 (30.0)	6 (26.1)	2 (33.3)
Severe	11 (16.2)	1 (3.5)	5 (50.0)	1 (4.3)	4 (66.7)

Demographic and participant-reported data describe *n* = 68 participants within the cohort. Presence of pain and pruritus was determined by mean numeric rating scale values (NRS) > 0. (EBS: EB simplex; DDEB: dominant dystrophic EB; RDEB: recessive dystrophic EB; JEB: junctional EB; SD: standard deviation; IQR: Interquartile range (25th–75th percentage)). (*/**) represent significant differences (Bonferroni adjusted) between two variables (*p* < 0.05).

**Table 2:** (a) Number of current systemic and local pharmacologic treatments for pain and/or pruritus, stratified by EB type. (b) Number of previous systemic pharmacologic treatments for pain and/or pruritus, stratified by EB type. (c) Proportion of participants reporting recreational drug-use and effects on EB. Participants described the effects of any indicated recreational drugs related to their “life with EB”. More than one could be reported.

(a) Treatment Indication	Drug Class	Number of Participants (%)	Number of (Simultaneous) Treatments	Total Count (%) (*n* = 68]	Count by EB-subtype			
					EBS (%) (*n* = 29)	DDEB (%) (*n* = 23)	RDEB (%) (*n* = 6)	JEB (%) (*n* = 10)
Pain	Paracetamol	24 (35.1)	None	36 (52.9)	21 (72.4)	8 (34.8)	2 (33.3)	5 (50.0)
	NSAID	8 (11.8)	1	24 (35.3)	7 (24.1)	11 (47.8)	4 (66.7)	2 (20.0)
	Opioid	3 (4.4)	2	5 (7.4)	1 (3.4)	3 (13.0)	-	1 (10.0)
	Anti-epileptic	2 (2.9)	3	1 (1.5)	-	1 (4.3)	-	-
	CBM	1 (1.5)	4	1 (1.5)	-	-	-	1 (10.0)
			5	1 (1.5)	-	-	-	1 (10.0)
Pruritus	Antihistamine	14 (20.6)	None	51 (75.0)	24 (82.8)	15 (65.2)	4 (66.7)	8 (80.0)
	Calcineurin Inhibitor	3 (4.4)	1	14 (20.6)	5 (17.2)	5 (21.7)	2 (33.3)	2 (20.0)
	Corticosteroid	3 (4.4)	2	2 (2.9)	-	2 (8.7)	-	-
	5HT3-Antagonist	1 (1.5)	3	1 (1.5)	-	1 (4.3)	-	-
(b) Treatment Indication	Drug Class	Number of Participants (%)	Number of Treatments Previously Used	Total Count (%) [*n* = 68]	Count by EB-subtype			
					EBS (%) (*n* = 29)	DDEB (%) (*n* = 23)	RDEB (%) (*n* = 6)	JEB (%) (*n* = 10)
Pain and Pruritus	Paracetamol	55 (80.9)	None	25 (36.8)	11 (37.9)	10 (43.5)	2 (33.3)	2 (20.0)
	NSAID	46 (67.6)	1	6 (8.8)	2 (6.9)	3 (13.0)	-	1 (10.0)
	Opioid	28 (41.2)	2	9 (13.2)	4 (13.8)	3 (13.0)	1 (16.7)	1 (10.0)
	Antihistamine	25 (36.8)	3	11 (16.2)	5 (17.2)	4 (17.4)	1 (16.7)	1 (10.0)
	Steroid	16 (23.5)	4	9 (13.2)	4 (13.8)	1 (4.3)	1 (16.7)	3 (30.0)
	Benzodiazepine	13 (19.1)	5	4 (5.9)	2 (6.9)	-	1 (16.7)	1 (10.0)
	CBM	12 (17.6)	6	1 (1.5)	-	1 (4.3)	-	-
	Antidepressant	10 (14.7)	7	3 (4.4)	1 (3.4)	1 (4.3)	-	1 (10.0)
	Anti-epileptic	4 (5.9)						
	Ketamine	1 (1.5)						
(c) Type of Recreational Drug	Number of Participants (%)		Effect on EB					
			EB Symptom Reduction	EB Symptom Worsening		Ability to Relax	No effect on EB	Missing
			n (%)					
Alcohol	49 (72.1)		7 (14.3)	4 (8.2)		4 (8.2)	6 (12.2)	28 (57.1)
Tobacco	12 (17.6)		-	-		5 (41.7)	3 (25.0)	4 (30.0)
Cannabis	8 (11.8)		5 (62.5)	1 (12.5)		3 (37.5)	1 (12.5)	-
Other drugs	1 (1.5)		-	-		-	1 (100.0)	-
Does not use	14 (20.6)		-					

**Table 3:** Patient benefit index.

		n	Median (IQR)	Range (Min–Max)	PBI ≥ 1 * (%)
(a) PBI	Global	68	1.34 (0.68–2.58)	0.00–4.00	60.3
	EBS	29	0.96 (0.59–2.20)	0.00–3.92	48.3
	RDEB	6	1.35 (0.43–2.34)	0.31–3.52	66.7
	DDEB	23	1.61 (0.80–2.93)	0.00–4.00	60.9
	JEB	10	2.28 (0.84–2.79)	0.32–3.81	80.0
	Male	30	1.41 (0.83–2.86)	0.00–4.00	60.0
	Female	38	1.31 (0.61–2.38)	0.00–4.00	60.5
(b) PBI subscales	Reducing Social Impairments	45	1.43 (0.37–2.67)	0.00–4.00	60.0
	Reducing Psychological Impairments	45	2.00 (0.79–3.00)	0.00–4.00	73.3
	Reducing Impairments due to Therapy	39	1.00 (0.50–3.00)	0.00–4.00	53.8
	Reducing Physical Impairments	63	1.55 (0.63–3.00)	0.00–4.00	71.4
	To have Confidence in Healing	41	1.33 (1.00–3.00)	0.00–4.00	82.9

Results are presented as median values, interquartile ranges and proportion of participants achieving a score ≥ 1. (a) PBI is stratified by subtype and sex. (b) PBI subscale scores are represented similarly. IQR: Interquartile range (25th and 75th percentiles). Additional information can be found in the supporting information available online (Table S2, Figure S1). * A PBI score ≥1 meant that a minimal clinically important difference (MCID) could be assumed.

## Data Availability

The data presented in this study are available in the manuscript.

## Supplementary Materials

The following are available online at https://www.mdpi.com/article/10.3390/jcm10245836/s1, Table S1: Patient Benefit Index (PBI) and PBI subscales differentiated by epidermolysis bullosa (EB) type. Table S2: Patient-rated importance of needs in all EB types from the Patient Needs Questionnaire (PNQ) (n = 68), and patient-rated importance (percentage) of needs using the PNQ, stratified by EB type. Table S3: Correlation scores of patient-reported outcomes (pain score; pruritus score; self-reported severity) and demographic data (age at time of inclusion; time since diagnosis) with 25 patient needs questionnaire (PNQ) items. Figure S1: PBI subscale scores by EB-type.
