# Supplementary material for: Identifying Epidermolysis Bullosa Patient Needs and Perceived Treatment Benefits: An Explorative Study Using the Patient Benefit Index
Source: J Clin Med. 2021 Dec 13;10(24):5836. doi: 10.3390/jcm10245836 (PMC8709493; doi:10.3390/jcm10245836)

Supporting information:

Table S1: Patient Benefit Index (PBI) and PBI subscales differentiated by epidermolysis bullosa (EB) type

Table S2: Patient-rated importance of needs in all EB types from the Patient Needs Questionnaire (PNQ) (n=68), and patient-rated importance (percentage) of needs using the PNQ, stratified by EB type

Table S3: Correlation scores of patient-reported outcomes (pain score; pruritus score; self-reported severity) and demographic data (age at time of inclusion; time since diagnosis) with 25 patient needs questionnaire (PNQ) items

Figure S1: Patient Benefit Index (PBI) subscale scores by EB type

Table S1 Patient Benefit Index (PBI) and PBI subscales differentiated by epidermolysis bullosa (EB) type.

SD: standard deviation, IQR: interquartile range (25th to 75th percentiles), JEB: junctional EB, EBS: EB simplex, RDEB: recessive dystrophic EB, DDEB: dominant dystrophic EB

		All *n* = 68	EBS *n* = 29	DDEB *n* = 23	RDEB *n* = 6	JEB *n* = 10
**Patient Benefit Index**	Mean	1.64	1.39	1.84	1.49	1.97
	SD	1.12	1.05	1.16	1.17	1.97
	Median	1.34	0.96	1.61	1.35	2.28
	Percentile 25	0.68	0.59	0.80	0.43	0.84
	Percentile 75	2.55	2.20	2.93	2.24	2.79
**Reducing Social Impairments**	Mean	1.57	1.20	1.63	1.73	2.09
	SD	1.25	1.18	1.33	1.32	1.21
	Median	1.43	0.83	1.57	2.33	2.00
	Percentile 25	0.40	0.25	0.43	0.67	1.00
	Percentile 75	2.67	2.00	3.00	2.67	3.00
**Reducing Psychological Impairments**	Mean	1.98	1.51	2.42	1.67	2.38
	SD	1.33	1.17	1.34	2.08	1.27
	Median	2.00	1.00	2.82	1.00	3.00
	Percentile 25	0.80	0.75	1.21	0.00	1.00
	Percentile 75	3.00	2.50	3.44	4.00	3.00
**Reducing Impairments Due To Therapy**	Mean	1.53	1.18	1.38	1.75	2.24
	SD	1.38	1.18	1.32	1.68	1.57
	Median	1.00	0.81	0.88	1.00	2.60
	Percentile 25	0.50	0.50	0.25	0.75	0.75
	Percentile 75	3.00	1.75	2.51	3.00	3.60
**Reducing Physical Impairments**	Mean	1.78	1.40	2.20	1.43	2.06
	SD	1.33	1.23	1.42	1.34	1.19
	Median	1.55	1.00	2.04	1.35	1.86
	Percentile 25	0.63	0.25	1.00	0.00	1.29
	Percentile 75	3.00	2.12	3.40	2.67	3.00
**Having Confidence In Healing**	Mean	1.86	1.39	2.27	2.17	2.06
	SD	1.25	1.06	1.26	1.40	1.51
	Median	1.33	1.00	2.40	2.00	2.17
	Percentile 25	1.00	1.00	1.20	1.17	1.00
	Percentile 75	3.00	1.50	3.29	3.17	3.00

Table S2 A) Patient-rated importance of needs in all EB types from the Patient Needs Questionnaire (PNQ) (n=68). **B**) Patient-rated importance (percentage) of needs using the PNQ, stratified by EB type

A						B			
PBI Subscale	Patient Needs Questionairre (PNQ) item	mean (SD)	median (IQR)*	Does apply: n (%)	Proportion of high importance: n (%)**	Percentage (%) proportion of high importance by EB type			
						EBS (n=29)	*DDEB (n=23)*	*RDEB (n=6)*	*JEB (n=10)*
Having Confidence in Healing	To find a clear diagnosis and therapy	1.57 (1.65)	1 (0-3)	41 (60.3)	28 (41.2%)	41.4	30.4	66.7	50.0
	To have no fear that the disease will progress	1.79 (1.71)	2 (0-4)	48 (70.6)	27 (39.7%)	31.0	30.4	66.7	70.0
	To have confidence in the therapy	1.41 (1.63)	0 (0-3)	37 (54.4)	26 (38.2%)	27.6	39.1	66.7	50.0
Reducing Impairments due to Therapy	To have to spend less time with daily care	1.29 (1.61)	0 (0-3)	38 (55.9)	21 (30.9%)	13.8	30.4	83.3	50.0
	To have fewer out-of-pocket treatment costs	1.35 (1.62)	0 (0-3)	44 (64.7)	21 (30.9%)	17.2	26.1	83.3	50.0
	To experience fewer side-effects	1.00 (1.42)	0 (0-2)	34 (50.0)	12 (17.6%)	17.2	8.7	66.7	10.0
	To be less dependent on doctor and clinic visits	0.84 (1.35)	0 (0-1.5)	31 (45.6)	12 (17.6%)	6.9	17.4	50.0	30.0
Reducing Physical Impairments	To be healed of all skin alterations	2.63 (1.49)	3 (2-4)	58 (85.3)	42 (61.8%)	41.4	73.9	100.0	70.0
	To be free of itching	2.41 (1.57)	3 (1-4)	57 (83.8)	41 (60.3%)	48.3	69.6	100.0	50.0
	To be free of pain	2.43 (1.64)	3 (0.5-4)	51 (75.0)	40 (58.8%)	69.0	34.8	83.3	70.0
	To no longer have a burning sensation on the skin	1.85 (1.68)	2 (0-4)	42 (61.8)	31 (45.6%)	44.8	34.8	66.7	60.0
	To be able to sleep better	1.43 (1.65)	0 (0-3)	39 (57.8)	24 (35.3%)	24.1	26.1	83.3	60.0
Reducing Psychological Impairments	To be able to lead a normal everyday life	2.03 (1.74)	3 (0-4)	46 (67.6)	35 (51.5%)	48.3	47.8	50.0	70.0
	To be able to engage in normal leisure activities	1.87 (1.66)	2 (0-3)	45 (66.2)	31 (45.6%)	48.3	39.1	50.0	50.0
	To gain in joy of living	1.46 (1.69)	0 (0-3)	37 (54.4)	24 (35.3%)	31.0	30.4	33.3	60.0
	To feel less depressed	1.29 (1.61)	0 (0-3)	34 (50.0)	21 (30.9%)	24.1	21.7	83.3	40.0
	To be more capable in daily life	1.65 (1.75)	1 (0-3.5)	39 (57.4)	28 (41.2%)	34.5	30.4	66.7	70.0
Reducing Social Impairments	To dare to show oneself more	1.59 (1.67)	1 (0-3)	42 (61.8)	26 (38.2%)	27.6	43.5	50.0	50.0
	To be able to have a normal working life	1.31 (1.67)	0 (0-3)	33 (48.5)	23 (33.8%)	31.0	26.1	83.3	30.0
	To be less burdened in partnership	1.26 (1.63)	0 (0-3)	36 (52.9)	20 (29.4%)	27.6	26.1	50.0	30.0
	To be able to have a normal sex life	1.10 (1.59)	0 (0-2.5)	31 (45.6)	17 (25.0%)	10.3	26.1	66.7	40.0
	To be less of a burden to relatives and friends	1.21 (1.47)	0.5 (0-2.5)	38 (55.9)	17 (25.0%)	17.2	17.4	83.3	30.0
	To be able to have more contact with other people	0.93 (1.44)	0 (0-2)	31 (45.6)	14 (20.6%)	10.3	17.4	66.7	30.0
Unspecified	To get better skin quickly	2.68 (1.43)	3 (2-4)	60 (88.2)	44 (64.7%)	58.6	60.9	83.3	80.0
	To regain control of the disease	1.71 (1.69)	1 (0-3)	45 (66.2)	28 (41.2%)	34.5	34.8	66.7	60.0

* Interquartile range, 25th-75th percentile; ** PNQ items reported to be *quite* or *very* important.

Table S3: Correlation scores of patient-reported outcomes (pain score; pruritus score; self-reported severity) and demographic data (age at time of inclusion; time since diagnosis) with 25 patient needs questionnaire (PNQ) items

	**Age at time of inclusion**		**Time since diagnosis**		**Self-reported severity**		**Pain score**		**Pruritus score**	
	Coefficient	Confidence Interval	Coefficient	Confidence Interval	Coefficient	Confidence Interval	Coefficient	Confidence Interval	Coefficient	Confidence Interval
To get better skin quickly	-0.017	[-0.258 - 0.250]	-0.167	[-0.410 - 0.097]	***0.325**	[0.108 - 0.508]	***0.385**	[0.147 - 0.608]	-0.001	[-0.249 - 0.243]
To be healed of all skin alterations	0.104	[-0.177 - 0.354]	-0.086	[-0.307 - 0.142]	***0.325**	[0.093 - 0.531]	***0.363**	[0.095 - 0.578]	0.057	[-0.201 - 0.301]
To be free of itching	0.100	[-0.142 - 0.354]	0.184	[-0.067 - 0.416]	***0.335**	[0.116 - 0.538]	0.232	[-0.021 - 0.471]	***0.503**	[0.289 - 0.671]
To be free of pain	-0.095	[-0.332 - 0.143]	-0.041	[-0.285 - 0.215]	***0.381**	[0.178 - 0.545]	***0.367**	[0.151 - 0.574]	0.045	[-0.185 - 0.280]
To be able to lead a normal everyday life	-0.053	[-0.319 - 0.203]	0.030	[-0.225 - 0.281]	***0.283**	[0.038 - 0.482]	***0.434**	[0.210 - 0.625]	0.044	[-0.170 - 0.292]
To no longer have a burning sensation on the skin	0.205	[-0.058 - 0.430]	-0.079	[-0.349 - 0.194]	***0.432**	[0.197 - 0.624]	***0.498**	[0.284 - 0.664]	0.098	[-0.142 - 0.357]
To be able to engage in normal leisure activities	-0.011	[-0.233 - 0.211]	-0.006	[-0.278 - 0.258]	***0.391**	[0.177 - 0.564]	***0.427**	[0.175 - 0.614]	-0.036	[-0.266 - 0.194]
To find a clear diagnosis and therapy	0.200	[-0.061 - 0.440]	0.096	[-0.163 - 0.367]	***0.382**	[0.154 - 0.567]	***0.398**	[0.163 - 0.598]	-0.086	[-0.319 - 0.160]
To be more capable in daily life	-0.101	[-0.374 - 0.174]	0.071	[-0.178 - 0.336]	***0.513**	[0.298 - 0.672]	***0.415**	[0.160 - 0.627]	0.036	[-0.199 - 0.302]
To regain control of the disease	0.026	[-0.228 - 0.271]	-0.137	[-0.361 - 0.105]	***0.428**	[0.182 - 0.622]	***0.356**	[0.089 - 0.591]	0.056	[-0.186 - 0.285]
To have no fear that the disease will progress	***0.267**	[0.045 - 0.451]	-0.024	[-0.285 - 0.249]	***0.327**	[0.077 - 0.542]	***0.320**	[0.076 - 0.555]	0.017	[-0.240 - 0.275]
To have confidence in the therapy	0.195	[-0.670 - 0.440]	0.089	[-0.171 - 0.375]	***0.439**	[0.196 - 0.641]	***0.443**	[0.238 - 0.611]	-0.027	[-0.267 - 0.212]
To dare to show oneself more	0.094	[-0.162 - 0.329]	0.002	[-0.239 - 0.227]	0.210	[-0.042 - 0.443]	***0.304**	[0.066 - 0.514]	0.054	[-0.177 - 0.295]
To be able to sleep better	0.088	[-0.147 - 0.302]	0.134	[-0.108 - 0.337]	***0.550**	[0.343 - 0.731]	***0.494**	[0.288 - 0.665]	***0.397**	[0.180 - 0.590]
To gain joy of living	0.106	[-0.145 - 0.341]	0.063	[-0.190 - 0.324]	***0.488**	[0.262 - 0.678]	***0.510**	[0.317 - 0.660]	0.009	[-0.235 - 0.212]
To be able to have a normal working life	-0.065	[-0.298 - 0.169]	0.034	[-0.247 - 0.321]	***0.418**	[0.107 - 0.179]	***0.331**	[0.099 - 0.536]	-0.022	[-0.270 - 0.226]
To have to spend less time with daily care	0.131	[-0.103 - 0.342]	0.222	[-0.052 - 0.460]	***0.544**	[0.339 - 0.718]	***0.426**	[0.207 - 0.619]	0.109	[-0.146 - 0.335]
To feel less depressed	0.035	[-0.206 - 0.285]	0.115	[-0.159 - 0.362]	***0.636**	[0.443 - 0.801]	***0.431**	[0.209 - 0.619]	0.017	[-0.254 - 0.255]
To have fewer out of pocket treatment costs	0.021	[-0.235 - 0.264]	0.226	[-0.013 - 0.450]	***0.539**	[0.330 - 0.694]	***0.373**	[0.146 - 0.561]	0.161	[-0.089 - 0.414]
To be less burdened in partnership	-0.011	[-0.255 - 0.229]	0.024	[-0.241 - 0.285]	***0.333**	[0.111 - 0.523]	***0.310**	[0.099 - 0.504]	0.101	[-0.143 - 0.326]
To be able to have a normal sex life	0.123	[-0.102 - 0.354]	0.241	[-0.022 - 0.488]	***0.476**	[0.239 - 0.671]	***0.354**	[0.120 - 0.553]	***0.231**	[0.017 - 0.437]
To be less of a burden to relatives and friends	-0.135	[-0.388 - 0.111]	0.177	[-0.091 - 0.418]	***0.556**	[0.357 - 0.728]	***0.327**	[0.066 - 0.552]	-0.024	[-0.258 - 0.208]
To be able to have more contact with other people	0.084	[-0.184 - 0.329]	0.228	[-0.002 - 0.124]	***0.493**	[0.223 - 0.704]	***0.266**	[0.021 - 0.493]	-0.093	[-0.324 - 0.110]
To experience fewer side-effects	0.175	[-0.059 - 0.419]	***0.286**	[0.028 - 0.504]	***0.332**	[0.086 - 0.541]	***0.267**	[0.015 - 0.477]	0.094	[-0.147 - 0.318]
To be less dependent on doctor and clinic visits	0.117	[-0.113 - 0.351]	***0.282**	[0.027 - 0.510]	***0.525**	[0.289 - 0.706]	***0.413**	[0.209 - 0.599]	0.184	[-0.054 - 0.405]

*__Correlation is significant at the 0.05 level (2-tailed).__

Degrees of correlation: 0.000-0.299 (negligible), 0.300-0.499 (low), 0.500-0.699 (moderate), 0.700-1.00 (high)

Figure S1: Patient Benefit Index (PBI) subscale scores by EB type

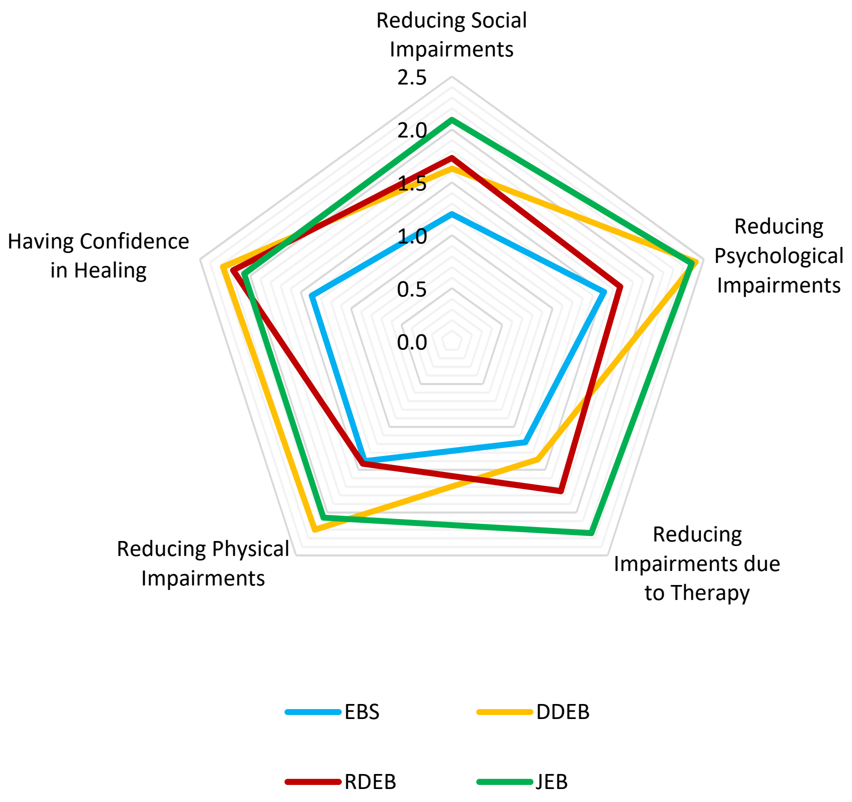

Supplement: Supplementary file 1 [file jcm-10-05836-s001.zip › jcm-1477345-supplementary.pdf]
